# Multi-method characterization of neurophysiological and biological stress responses in surgical teams during real surgical procedures

**DOI:** 10.3389/fnrgo.2026.1702748

**Published:** 2026-02-18

**Authors:** Vincenzo Ronca, Lidia Castagneto Gissey, Maria Irene Bellini, Alessandra Iodice, Valentina Sada, Emilia Sbardella, Ludovica Vincenzi, Pietro Aricò, Gianluca Di Flumeri, Andrea Giorgi, Alessia Vozzi, Rossella Capotorto, Fabio Babiloni, Giovanni Casella, Gianluca Borghini

**Affiliations:** 1 Department of Computer, Control, and Management Engineering “Antonio Ruberti”, Sapienza University of Rome, Rome, Italy; 2 BrainSigns srl, Rome, Italy; 3 Department of Surgery, Policlinico Umberto I, Sapienza University of Rome, Rome, Italy; 4 Department of Experimental Medicine, Sapienza University of Rome, Rome, Italy; 5 Department of Molecular Medicine, Sapienza University of Rome, Rome, Italy; 6 Department of Anatomical, Histological, Forensic and Orthopedic Sciences, Sapienza University of Rome, Rome, Italy; 7 Department of Computer Science, Hangzhou Dianzi University, Hangzhou, China; 8 Department of Physiology and Pharmacology “Vittorio Erspamer”, Sapienza University of Rome, Rome, Italy

**Keywords:** biological samples, electroencephalography, human factors, neurophysiological, stress

## Abstract

**Purpose:**

The surgical operating room is a high-stakes environment where stress can impact performance and patient safety. While hormonal and neurophysiological markers are established stress indicators, integrative studies in real-world surgical settings are scarce. This study aimed to provide a comprehensive, multimodal characterization of stress in surgical teams during live operations, comparing neurophysiological, biological, and behavioral responses across different levels of expertise and surgical phases. The goal was to validate a multi-method approach and identify objective markers for monitoring stress in real-time.

**Method:**

Surgical teams, each composed of four members, were categorized as “Expert” or “Novice” based on the lead surgeon's experience. All teams performed a standardized inguinal hernia repair. Continuous electroencephalography (EEG) and electrodermal activity (EDA) were recorded throughout the procedure to derive stress indices. Blood samples were collected pre- and post-surgery to measure Adrenocorticotropic Hormone (ACTH) and cortisol levels. Subjective stress was assessed via questionnaires, and team performance was quantified using a Combined Behavioral Teamwork Index (CBTI) based on surgical time, materials used, and patient outcomes.

**Finding:**

Neurophysiological data showed that the EEG-based stress index was significantly higher in Novice surgeons compared to Experts, particularly during the final and most demanding phase of the surgery (*p* = 0.008). This effect was most pronounced for the lead Novice surgeon (*p* = 0.01). Similarly, the EDA-based stress index was higher overall in Novices (*p* = 0.02). Post-surgery, ACTH levels increased significantly in Novices while decreasing in Experts (*p* = 0.008), indicating a sustained endocrine stress response in the less experienced group. Strong positive correlations were found between the EEG-stress index and both ACTH levels (*R* = 0.67) and subjective stress (*R* = 0.63), validating the multimodal assessment.

**Conclusion:**

This study demonstrates that a multimodal approach can effectively characterize stress dynamics in a real-world surgical environment. The EEG-derived metric emerged as the most sensitive indicator, capable of discriminating stress levels with high temporal and role-specific precision. Novice surgeons exhibit significantly greater neurophysiological and endocrine stress responses, underscoring the need for targeted support and advanced training protocols. These findings lay the groundwork for developing real-time, objective stress monitoring systems to enhance surgical performance, training, and patient safety.

## 1 Introduction

### 1.1 Stress in surgery rooms

The surgical operating room is a highly demanding environment, where clinical outcomes often depend on the ability of the surgical team to maintain optimal performance under stress. Time pressure, complex decision-making, intraoperative complications, and team coordination all contribute to heightened cognitive and emotional demands (Borghini et al., 2020c). Several studies have shown that excessive or poorly managed stress can impair attention, increase error rates, and negatively affect patient safety (Tam et al., 2024; Arora et al., 2010; Wetzel et al., 2006). For this reason, a growing number of studies have attempted to monitor the stress of surgical personnel in real settings with the aim of informing training programs, improving safety protocols, and developing adaptive technologies (Dimou et al., 2016). However, real-time, multi-parametric stress assessments remain scarce, and most of the current literature is still limited to simulated or controlled environments (Stefanidis et al., 2006; Gurusamy et al., 2009). The aim of the study was not to artificially induce stress, but to document the naturally occurring neurophysiological, autonomic, and endocrine stress responses that emerge during a standard surgical procedure performed under real clinical conditions.

### 1.2 Stress biological correlates

The biological response to stress is mediated primarily by the hypothalamic–pituitary–adrenal (HPA) axis, which orchestrates the secretion of adrenocorticotropic hormone (ACTH) and cortisol. These hormones are widely recognized as sensitive markers of both acute and chronic stress. While several studies have evaluated these hormones in surgical contexts (Arora et al., 2010; Kumari et al., 2021; McEwen, 2017; Silva et al., 2021), other high-stakes domains have similarly relied on these biomarkers to investigate stress in ecologically valid scenarios. For example, Buchanan and Lovallo (2001) demonstrated increased salivary cortisol during public speaking tasks, emphasizing the hormone's responsiveness to psychosocial stressors. Schommer et al. (2003) showed that both ACTH and cortisol levels rose significantly in air traffic controllers during high workload periods. In the military domain, Morgan et al. (2000) studied soldiers undergoing Survival, Evasion, Resistance, and Escape (SERE) training, observing robust increases in ACTH and cortisol levels in response to simulated captivity. Similarly, Filaire et al. (2001) reported elevated cortisol levels in elite athletes before and after competition, correlating with both perceived anxiety and physical exertion. These findings collectively underscore the value of ACTH and cortisol as endocrine correlates of stress, not only in clinical or experimental settings but also in field environments involving physical or cognitive strain. However, studies that combine these biomarkers with real-time physiological measures such as EEG or EDA are still limited, particularly in team-based operational contexts like surgery. The role of prolactin as a marker of acute stress response was the subject of several studies. The Trier social stress test, a survey conducted by Lennartsson and Jonsdottir (2011), showed a significant rise in prolactin level, along with ACTH and cortisol levels, with a peak at +20 time point in all patients, without differences between men and women. The transient increase of prolactin levels has also been observed in real-life acute stress studies, performed by Schedlowski et al. (1992). Plasma levels of prolactin, cortisol, thyroid-stimulating hormone (TSH), and luteinizing hormone (LH) were studied in 12 experienced and 11 inexperienced military parachutists. Before and immediately after each jump, all patients underwent blood samples for plasma levels of cortisol, prolactin, TSH and LH. Prolactin levels were significantly increased after the jump in both groups, showing that jump experience did not interfere with stress-induced hormonal responses. These data have recently been challenged. Güneş et al. (2025) conducted a case-control study to assess whether transient hyperprolactinemia during medical procedures reflects psychological stress. In 91 patients, serial prolactin levels and validated anxiety measures showed no significant correlation, suggesting that mild, transient prolactin elevations are not necessarily stress-induced. Moreover, plasma free metanephrines, particularly normetanephrine, have been studied for their possible transient increase in response to acute physical or psychological stress. A study by Vage et al. (2025) found that during a controlled acute psychological stress, cortisol levels showed a significant increase in healthy individuals exposed to varying levels of psychological stress, while plasma metanephrine levels did not exhibit a consistent pattern of change, suggesting that they may not be as sensitive to acute psychological stress as cortisol.

### 1.3 Stress neurophysiological correlates

Neurophysiological signals offer unique insights into the real-time dynamics of stress, especially in environments where continuous and unobtrusive monitoring is critical. Among these, electroencephalography (EEG) has proven particularly valuable for its ability to reveal stress-related changes in cortical activity. Stress is typically associated with an increase in frontal-midline theta power and a suppression of alpha rhythms, reflecting increased cognitive control and reduced relaxation, respectively (Harmony, 2013). Similarly, electrodermal activity (EDA)—quantifying sympathetic nervous system activation through skin conductance (SC)—is a well-established indicator of emotional arousal. In non-medical but ecologically valid contexts, several studies have leveraged EEG and EDA to track stress. For instance, Borghini et al. (2022a) monitored EEG in pilots during flight simulation, finding increased theta and beta activity during emergency conditions. Sciaraffa et al. (2022a) applied EEG-based stress indices to air traffic controllers, demonstrating sensitivity to task difficulty and time-on-task effects. Matsumoto et al. (2021) recorded EDA and heart rate during realistic emergency simulations, revealing significant sympathetic activation during the most intense procedural stages. Similarly, Setz et al. (2010) found that EDA could predict mental stress levels in office workers exposed to time pressure and multitasking, confirming the utility of EDA even in cognitively demanding but less physically intense environments. In military simulations, Pope et al. (1995) developed a “neuroadaptive” training system using EEG to track and adapt to soldiers' stress and workload during virtual missions (Borghini et al., 2017). In the sports field (Borghini et al., 2020a; Dziembowska et al., 2016) assessed professional archers and found that EEG-derived frontal asymmetry correlated with anxiety and performance under pressure.

Despite this breadth of applications, few studies have combined EEG and EDA with hormonal markers in synchronized, longitudinal designs. Moreover, in team-based real-world environments such as surgery, where inter-individual variability and role-specific dynamics are important, such multimodal monitoring is still underdeveloped. A recent systematic review by Torkamani-Azar et al. (2022) provides a comprehensive overview of sensor-based methods for mental stress assessment in surgical settings, highlighting both the technological advancements and the lack of multimodal, real-world investigations.

### 1.4 Objective of the study

While both hormonal and neurophysiological markers have been independently validated as stress indicators, there is a lack of integrative, role-specific, and real-world studies exploring how stress unfolds across surgical phases and professional experience levels. The present study aims to address this gap by conducting a comprehensive, multimodal characterization of stress responses in general surgical teams during real-time surgical procedures in the operating room, led by either an attending surgeon or a surgical resident. Although the primary comparison of expertise levels was centered on the Operating Surgeon, stress responses were also quantified for all members of the surgical team (First Assistant, Second Assistant, and Scrub Nurse) to capture a complete and ecologically valid picture of intraoperative stress dynamics. EEG and EDA data were continuously recorded throughout surgical procedures, while blood samples for the evaluation of ACTH and cortisol levels were collected *pre-* and *post-*surgery. The goals of the study were to:

- Quantify how EEG- and EDA-derived stress indices evolved across three phases of the surgery.
- Investigate how biological stress markers (i.e., ACTH, cortisol) varied between pre- and post-surgical states in both Novice and Expert groups.
- Compare stress profiles across professional roles to identify risks of stress overload.
- Validate the multi-method approach for stress evaluation through time dynamics comparison of the proposed stress-related indicators.

By combining multiple physiological and endocrine markers in real surgical environments, this study sought to provide a richer understanding of the temporal and role-specific stress patterns in operative medicine. These insights could inform future surgeon support systems, adaptive training frameworks, and stress management protocols aimed at enhancing surgical performance and safety.

## 2 Materials and methods

### 2.1 Experimental sample and design

The study was conducted at the “Policlinico Umberto I” Hospital, Sapienza University of Rome (Italy), involving surgical teams recruited from the Department of Surgery. The experiments were conducted following the principles outlined in the Declaration of Helsinki of 1975, as revised in 2008 and was approved by the Sapienza University of Rome Ethical Committee in Charge for the Sapienza University of Rome (protocol number: 0746/2023, approved on 20/09/2023). The informed consent was obtained from all the participants involved in the study. To respect the privacy of surgeons, only aggregate results were reported, and no results based on single identity analysis were presented, in accordance with the Regulation of the European Parliament 679/2016. Each team was composed of four members: the Operating Surgeon (*S*), First Assistant (*A*1), Second Assistant (*A*2), and Scrub Nurse (*N*). Teams were categorized based on the experience level of the Operating Surgeon into two groups: Expert (i.e. attending surgeon) and Novice (i.e. surgical resident). In total, eight complete surgical teams participated in the study (*N* = 32 participants), equally distributed into four Expert and four Novice teams. As part of the pre-operative screening, all participants confirmed that they had not consumed caffeine since waking on the day of surgery, in compliance with hospital fasting and stimulant avoidance guidelines. None of the participants were undergoing pharmacological treatments or taking medications known to affect autonomic, endocrine, or neurophysiological function.

All teams performed an inguinal hernia repair following the Lichtenstein technique (Messias et al., 2024), a standardized procedure selected for its suitability in allowing Novice surgeons to act as primary operators under supervision. The inguinal hernia repair was intentionally selected because it provides a standardized, reproducible sequence of steps suitable for both expert and novice surgeons. This ensures comparability across teams and enables less experienced surgeons to operate as primary surgeons under supervision, without introducing additional procedural stressors unrelated to expertise. Additionally, this procedure can be safely performed by Novice surgeons under appropriate supervision. This choice ensured that both Experts and Novices could act as primary operators within a comparable operative framework, enabling the formation of two parallel groups. Without selecting a procedure suitable for Novices, it would not have been possible to obtain two groups matched for operative responsibility while maintaining procedural standardization. Surgeons in the Expert group had completed at least 80 inguinal hernia repairs as lead surgeon and were listed in the National Specialist Register. In contrast, the Novice group included residents enrolled in a national general surgery residency program, with a minimum of 3 years of training and prior participation in at least 90 inguinal hernia repairs, including at least 40 cases as first assistant and 15 cases as primary operator under direct Expert supervision.

**Figure 1:**
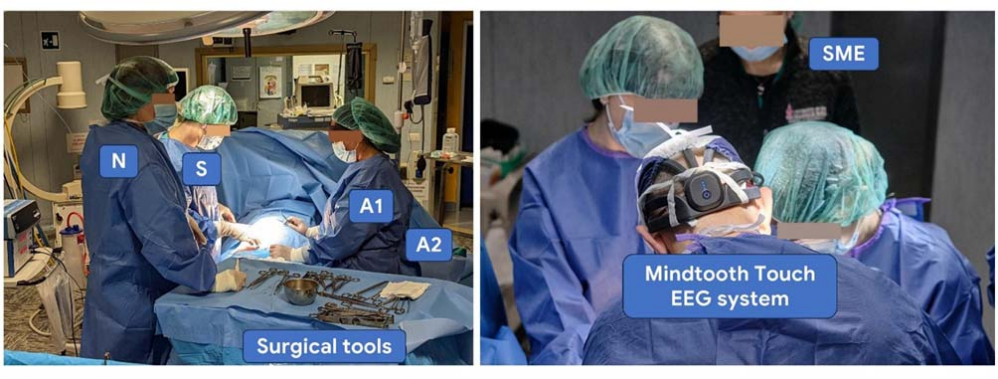
Experimental settings representing all the surgery roles and positions during the data collection. Each team was composed of four members: the operating surgeon (*S*), first assistant (*A*1), second assistant (*A*2), and scrub nurse (*N*).

All surgeries were conducted in a real clinical setting (Figure 1), under standard conditions and scheduled at a consistent time of day to minimize circadian effects on physiological parameters. The surgical procedure was divided into three predefined phases:

- Phase 1—from skin incision to spermatic cord identification and isolation.
- Phase 2—dissection of the hernia sac.
- Phase 3—mesh placement followed by fascia and skin closure.

While data were segmented into three predefined surgical phases, the primary analyses focused on overall between-group differences across the entire procedure. Phase-specific comparisons were conducted as exploratory analyses to characterize the temporal dynamics of stress responses, and were interpreted cautiously with appropriate control for multiple comparisons. A baseline recording was collected prior to the beginning of the surgical procedure, consisting of two 1-min sessions: one with eyes open, used to capture blink-related EEG patterns, and one with eyes closed, employed for the calibration of EEG spectral bands.

### 2.2 Subjective and behavioral data

Subjective data were collected during surgery by filling in different kinds of questionnaires. Such questionnaires were administered during and right after the end of the surgery to have a subjective assessment of stress for each member. More specifically, the Subject Matter Expert (SME) filled in the questionnaires every 5 min, at the end of each phase of the surgery, and at the end of the experiment. Right after the end of the surgery, the surgeons filled in a survey by which assessing their own stress. All answers were collected on a 5-point Likert scale. Additionally, the SME focused on several performance indicators while observing the surgical team. These indicators were determined at the beginning of the study in collaboration with various SMEs to differentiate an adequate performance from an excellent performance. Every 5 min, the SME rated the speed with which the surgeons were working, the coordination of the surgeons' movements and the level of intraoperative bleeding. After the end of each of the three main surgical phases, the SME recorded the number of materials used (i.e., fixators, retractors, forceps, retractors, needle holders, scissors, and gauzes). Finally, the SME assessed the accuracy and quality of mesh placement. The completion time associated with the entire surgery session was collected per each surgical team involved in the study. In this regard it has to be specified that a single Subject Matter Expert (SME), a senior surgeon with more than 10 years of operative experience, was responsible for observing and evaluating the surgical teams. The SME was blinded to the team's level of expertise (Expert vs. Novice) and underwent a dedicated calibration session before the start of data collection to ensure consistency in applying the observational criteria. The evaluation grid was specifically designed for this study to capture key behavioral dimensions such as coordination, communication, and intraoperative adaptability to stress—factors that complement rather than overlap with technical proficiency. Although validated tools such as the Objective Structured Assessment of Technical Skills (OSATS; Martin et al., 1997) are widely used for assessing individual technical skills, they are not intended to quantify team-level behavioral performance or stress-related responses. For this reason, the present SME-based evaluation focused on behavioral and teamwork-related indicators observed under natural surgical conditions, in accordance with non-technical skill frameworks proposed for the surgical domain (Yule et al., 2006; Flin et al., 2006).

Finally, to obtain a single team-level performance indicator, we defined the Combined Behavioral Teamwork Index (CBTI) as the average of three normalized components: surgical duration, Subject Matter Expert (SME) evaluations, and patient outcome. Importantly, all components were normalized within the present dataset (i.e., across the eight surgical teams included in this study) to avoid dependence on external normative datasets. Because CBTI is computed once per surgical team (and is therefore identical for all members within the same team), CBTI group comparisons were performed at the team level (*n* = 4 Expert teams vs. *n* = 4 Novice teams).

Let *t*_*i*_ be the total procedure duration for team *i*, *m*_*i*_ the total number of materials used by team *i*, and *o*_*i*_ the patient outcome score for team *i*. Patient outcome was collected on a discrete Likert scale ranging from 1 (worst) to 5 (best). Since lower duration and fewer materials reflect better performance, their normalized scores were inverted so that higher values always indicate better performance:


$$\begin{array}{l} {\;T}_{i}=1-\frac{t_{i}-\min\left(t\right)}{\max\left(t\right)-\min\left(t\right)}\\ M_{i}=1-\frac{m_{i}-\min\left(m\right)}{\max\left(m\right)-\min\left(m\right)}\\ {\;O}_{i}=\frac{o_{i}-1}{5-1} \end{array}$$

The CBTI for team *i* was then computed as:


$$\begin{array}{l} CBTI_{i}=\frac{T_{i}+M_{i}+O_{i}}{3} \end{array}$$

With this definition, *CBTI*_*i*_ϵ[0, 1], where higher values correspond to better overall team performance (shorter duration, lower material consumption, and better patient outcome). Previous research in surgical settings has emphasized the critical role of non-technical skills, including communication, leadership and teamwork, in influencing intra-operative team performance (Yule et al., 2006). Studies of simulated trauma teams suggest that shared mental models and behavioral teamwork skills significantly contribute to performance outcomes (Westli et al., 2010). Systematic reviews of teamwork assessment tools in surgery confirm that observable team behaviors (e.g., coordination, role clarity, resource management) are measurable and relate to efficiency and safety (Whittaker et al., 2015). Furthermore, ethnographic work in real operating rooms has highlighted how shared goals, knowledge and mutual respect underpin high-performing surgical teams (Tørring et al., 2019). In light of this evidence, we operationalized the CBTI to integrate key observable components of team design (efficiency, resource management and outcome quality). Higher CBTI values reflect better overall team performance.

### 2.3 Biological samples collection and analysis

Biological samples were collected at two standardized timepoints: *T*0 (pre-surgery), immediately before entering the sterile field and before skin incision, and *T*1 (post-surgery), immediately after completion of skin closure. At both timepoints, venous blood was collected for the assessment of ACTH and cortisol (primary endocrine outcomes). Additional biological matrices were collected as part of an exploratory panel (urine and saliva for cortisol; blood for prolactin and metanephrines); however, the main between-group analyses and endocrine results reported in this manuscript focus on ACTH and cortisol measured at *T*0 and *T*1.

The analysis of all collected samples was performed at the study center. Serum and spot urine cortisol were measured by radioimmunoassay, using Beckman Coulter reagents (ref. IM1841). The measurement range (from analytical sensitivity to the highest calibrator) is 8.60–2,000 nM. ACTH was measured by immunoradiometric assay, using Beckman Coulter reagents (ref. IM2030, B89463). We collected EDTA plasma samples, and we processed them as soon as possible by centrifugation at 2–8 °C. Due to the high ACTH degradation, plasma samples were stored at−18 °C unless the assay was done immediately. The measurement range (from analytical sensitivity to highest calibrator) is 0.31 to approximately 1,500 pg/ml. Serum metanephrines were measured using an Enzyme-Linked Immunosorbent Assay (ELISA) with DRG reagents (ref. EIA 4313). The measurement range (from analytical sensitivity to the highest calibrator) is 15.1–3,600 pg/ml. We collected EDTA plasma samples, and we processed them as soon as possible by centrifugation at 2–8 °C. Serum prolactin was measured by Chemiluminescent Microparticle Immunoassay (CMIA), using Abbott ARCHITECT reagents (ref. 7K76-25). The measurement range (from analytical sensitivity to the highest calibrator) is 0–200 ng/ml. We collected EDTA plasma samples, and we processed them as soon as possible by centrifugation at 2–8 °C.

### 2.4 Neurophysiological signals collection and analysis

#### 2.4.1 Electroencephalographic (EEG) stress assessment

EEG signals were recorded using the Mindtooth Touch digital monitoring system (BrainSigns srl, Rome, Italy), with a sampling frequency of 125 Hz. Eight water-based electrodes were positioned over frontal and parietal regions commonly associated with cognitive and mental state monitoring (Borghini et al., 2020c, 2022b; Di Flumeri et al., 2022; Ronca et al., 2022, 2025a; Simonetti et al., 2023; Vincenzo et al., 2025). Specifically, electrodes were placed at Afz, Af3, Af4, Af7, Af8, Pz, P3, and P4, referenced to the right mastoid and grounded at the left mastoid. Prior to data collection, electrode impedance was verified to be below 50 kΩ to ensure adequate signal quality (Sciaraffa et al., 2022a). Before analysis, the EEG data underwent a pre-processing phase aimed at identifying and correcting physiological and non-physiological artifacts unrelated to cerebral activity of interest (e.g., ocular movements, muscle activity, body motion). Signals were band-pass filtered between 2 and 30 Hz using a fifth-order Butterworth filter. Eye-blink artifacts were detected and corrected in real time using the o-CLEAN method (Di Flumeri et al., 2016a; Ronca et al., 2024a). Additional artifacts, including those from muscular activity and movements, were addressed using *ad-hoc* algorithms based on the EEGLAB toolbox (Delorme and Makeig, 2004). Following pre-processing, the Global Field Power (GFP) was calculated within specific frequency bands relevant for cognitive and mental state assessment. GFP was computed for Theta, Alpha, and Beta bands, offering a robust measure of the degree of cortical synchronization within these bands over the regions of interest (Di Flumeri et al., 2016b). GFP was chosen as the primary EEG metric due to its ability to capture spatially integrated neural activity over time. The computation followed the approach described by Vecchiato et al. (2014). Frequency bands were individualized based on each participant's Individual Alpha Frequency (IAF; Klimesch, 1999). The IAF was determined from a 60-s baseline recording acquired with participants' eyes closed, a condition that reliably enhances the alpha peak. Subsequently, EEG GFP features were extracted for each 1-s epoch using a Hanning window of matching length, yielding a frequency resolution of 1 Hz suitable for the analysis framework adopted. From the artifact-free EEG data, the GFP was then computed within the High Beta band (21–26 Hz), which was individually defined based on each participant's IAF (i.e., IAF + 11 Hz to IAF + 16 Hz). The stress neurometric was derived in accordance with established methods reported in the literature (Sciaraffa et al., 2022b):


$$\begin{array}{l} Stress_{EEG}=\;Beta\;High_{GFP}\left\{P_{3},\;P_{4}\right\} \end{array}$$

### 2.5 Physiological (EDA) stress assessment

Electrodermal activity (EDA) reflects changes in skin conductance (SC), where increases in skin conductance level (SCL) are widely recognized as reliable markers of human arousal (Eysenck et al., 2007). SCL is commonly used to assess anxiety in response to challenging situations or stress induced by perceived threats (Reinhardt et al., 2012; Borghini et al., 2020b). To avoid interference with manual actions, sterility constraints, and instrument handling during the surgical procedure, EDA sensors were placed on the hallux (big toe) and second toe of the non-dominant foot. This placement ensures high-quality conductance measurement while preventing disruptions to the operative workflow. EDA signals were pre-processed using a MATLAB-based pipeline. The data were first down-sampled from 64 Hz to 8 Hz and then filtered with a fifth-order Butterworth low-pass filter (cut-off frequency: 2 Hz) to eliminate high-frequency components unrelated to electrodermal activity, such as artifacts from motion or abrupt changes in electrode-skin contact pressure. These latter artifacts typically appear as brief, sharp peaks in the EDA signal. To address this, a two-step procedure combining automatic detection with expert visual inspection was implemented, and identified transients were corrected using piecewise cubic spline interpolation (Benedek and Kaernbach, 2010a). Subsequently, the signal was analyzed using Ledalab, an open-source MATLAB toolbox for EDA processing. The Continuous Decomposition Analysis (CDA) method (Benedek and Kaernbach, 2010b) was applied to separate the SCL and skin conductance response (SCR) components. For this study, the mean SCL during each experimental condition was used as the primary physiological index of stress.

### 2.6 Statistical analyses

The EEG-based stress index was subjected to normalization through the application of the z-score approach, referencing the data series to the eyes-open experimental condition, considered as the baseline for each participant. While the EDA-based stress index was normalized according to the minimum and maximum SCL value computed along the entire experimental session for each participant. A mixed within–between ANOVA design was initially considered for evaluating the interaction between expertise level (Expert vs. Novice) and surgical phases. However, preliminary tests revealed violations of normality and sphericity assumptions, combined with the limited sample size of each group, which would have compromised the reliability of mixed-model estimates. For this reason, we adopted a more conservative analytic strategy based on pairwise comparisons with appropriate parametric or non-parametric tests, depending on data distribution, as recommended for small sample, real-world datasets. As a preliminary step, the Shapiro–Wilk test (Shapiro and Wilk, 1965) was selected to determine the normality of the distribution related to each of the considered statistical feature. In case of normal distributions, the parametric ANOVA was selected for comparing the two experimental groups (i.e., Expert vs. Novices) along the three experimental phases. If distributions' normality was not confirmed by the Shapiro-Wilk test, the Friedman test was performed. *Post-hoc* comparisons were performed using either independent-samples Student's *t*-tests (for normally distributed variables) or Mann–Whitney *U*-tests (for non-normal distributions). Within-group pre–post comparisons (ACTH and cortisol) used paired *t*-tests or Wilcoxon signed-rank tests as appropriate. Additionally, the repeated measures correlation analysis (Bakdash and Marusich, 2017) was performed to investigate the temporal dynamic coherence between the EEG-based and the EDA-based stress indexes and the subjective and behavioral measurements collected from the surgical team, both at the single-participant level and in the entire group. To control for potential Type I errors arising from multiple comparisons, the False Discovery Rate (FDR) correction (Benjamini–Hochberg procedure) was applied across multimodal analyses. Detailed statistics, including *n*, degrees of freedom, effect sizes, and 95% confidence intervals, are reported in Supplementary Table 1.

## 3 Results

The results have been organized in three different subsections, respectively *Subjective and Behavioral, Neurophysiological Assessment*, and *Biological Assessment*, to facilitate the reader. All the results are here shortly described, pointing out the relevant findings, while they will be commented in a more comprehensive view along the *Discussion* section.

For clarity, the specific statistical test applied to each analysis is explicitly reported in the Results text, while a complete overview of all tests, degrees of freedom, and effect sizes is provided in Supplementary Table 1.

### 3.1 Subjective and behavioral

Group differences in subjective and behavioral measures between Expert and Novice teams were assessed using an independent-samples Student's *t*-test. The analysis revealed notable differences between Expert and Novice surgical teams. The Combined Behavioral Teamwork Index (CBTI), which integrates surgical duration, Subject Matter Expert (SME) evaluations, and patient outcomes as a performance metric, was significantly higher in Expert teams (*t*(6) = 2.89, *p* = 0.02, effect size = 0.76; Supplementary Table 1), indicating superior overall performance. In terms of perceived stress, Novice surgeons reported a higher subjective stress levels on self-assessment questionnaires compared to Experts, although this difference did not reach statistical significance (Student's *t* = 1.81; *p* = 0.07, *r* = 0.31). Similarly, the stress levels evaluated by SMEs showed no significant difference between the two groups but followed a statistical trend consistent with self-reported stress (Student's *t* = 1.04; *p* = 0.09). Furthermore, patients tended to report greater combined discomfort, encompassing pain, mental, and physical discomfort, when operated on by Novice teams, though this difference also remained at a statistical trend level (Student's *t* = 1.03; *p* = 0.1). These results suggest that while objective measures of performance significantly favored the Experts, both subjective stress perception and patient-reported discomfort tended to be greater in association with Novice teams.

### 3.2 Neurophysiological assessment

**Figure 2:**
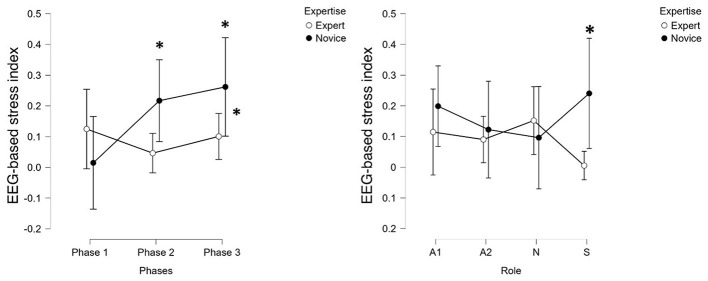
The statistical analysis revealed a significant increase of the EEG-based stress index during phase 3 only for Novice group (on the left), while the analysis performed along the whole experiment (on the right) showed that a significant difference between groups only for the first surgeons (i.e., *S*). *Indicates statistical significance (*p* < 0.05).

Differences in the EEG-derived stress index between Expert and Novice groups were assessed using a one-way between-subjects ANOVA. The neurophysiological analysis revealed statistical differences in stress responses between the two surgeons' groups. The EEG-derived stress index, based on beta-band activity over parietal regions, was significantly higher in Novices compared to Experts across the entire surgical procedure (*F*(1, 30) = 8.21, *p* = 0.008, η^2^ = 0.21; Supplementary Table 1) and specifically during Phase 3 (Figure 2, left). While Novices consistently exhibited elevated stress levels in Phase 2 and Phase 3 (*post-hoc t*(30) = 2.72, *p* = 0.010, *r* = 0.45; Supplementary Table 1). These phase-specific group differences are reported as secondary analyses aimed at describing within-procedure stress dynamics. Role-specific differences in EEG-derived stress (First Surgeon only) were assessed using a one-way between-subjects ANOVA. In this regard, Novice first surgeons (*S*) showed a significantly higher neurophysiological stress index compared to their Expert counterparts (*F*(1, 14) = 7.55, *p* = 0.011, η^2^ = 0.35; Supplementary Table 1), with a similar trend observed for the first assistant (*A*1; Figure 2, right).

**Figure 3:**
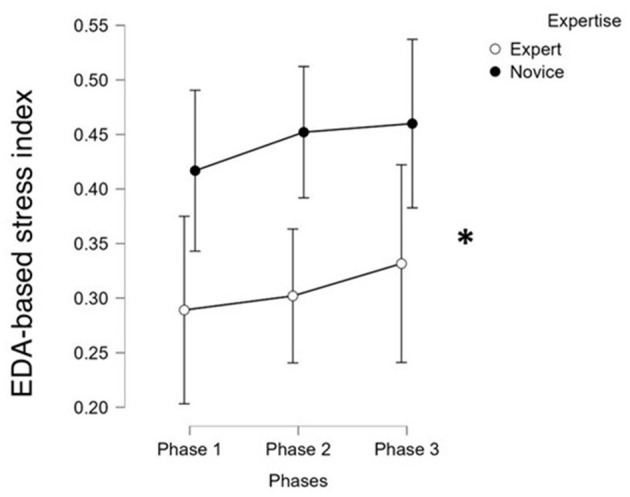
The statistical analysis performed on the EDA-based stress index highlighted only a significant difference between novice and experts along the whole experimental session. *Indicates statistical significant comparisons.

Group differences in EDA-derived stress were assessed using a one-way between-subjects ANOVA. Such a stress index, reflecting autonomic arousal via skin conductance level (SCL), was also significantly higher in novices overall (*F*(1, 30) = 5.87, *p* = 0.020, η^2^ = 0.16; Supplementary Table 1), particularly during Phase 2 (*t*(30) = 2.32, *p* = 0.029, *r* = 0.39; Supplementary Table 1; Figure 3). However, the EDA index did not significantly vary across roles (*p* > 0.2).

Importantly, significant positive correlations emerged between the EEG-derived stress index and both subjective stress perception (*R* = 0.63, *p* < 10^−3^; Supplementary Figure 1) and the EDA-derived stress index (*R* = 0.51, *p* < 10^−5^; Supplementary Figure 2), highlighting temporal consistency between neurophysiological, autonomic, and perceived stress dynamics throughout the surgery.

### 3.3 Biological assessment

**Figure 4:**
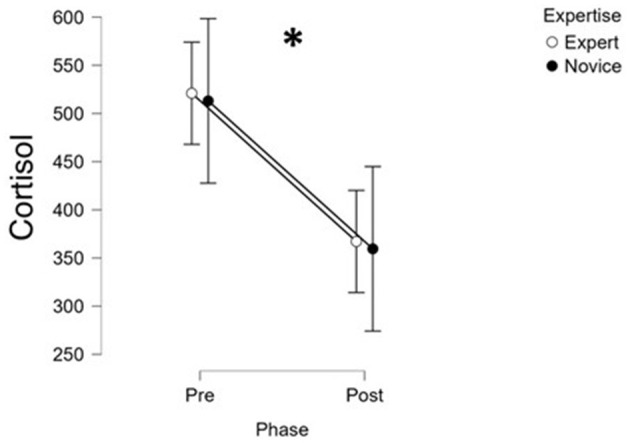
The statistical analysis performed on the cortisol biological parameter revealed a significant decrease in post-surgery for both Novice and Experts groups. *Indicates statistical significant comparisons.

ACTH levels were analyzed using a mixed-design ANOVA with Group (Expert vs. Novice) as a between-subject factor and Time (pre vs. post) as a within-subject factor, while changes in cortisol levels from pre- to post-surgery were assessed using paired-samples *t*-tests. The analysis of biological stress markers revealed distinct patterns for ACTH and cortisol across the surgical teams. Cortisol levels showed a significant decrease from pre- to post-surgery (i.e., from *T*0 to *T*1) in both Expert and Novice groups (*t*(31) = 2.89, *p* = 0.007, *r* = 0.46; Supplementary Table 1; Figure 4), indicating a general reduction in HPA axis activation following the completion of the procedure.

**Figure 5:**
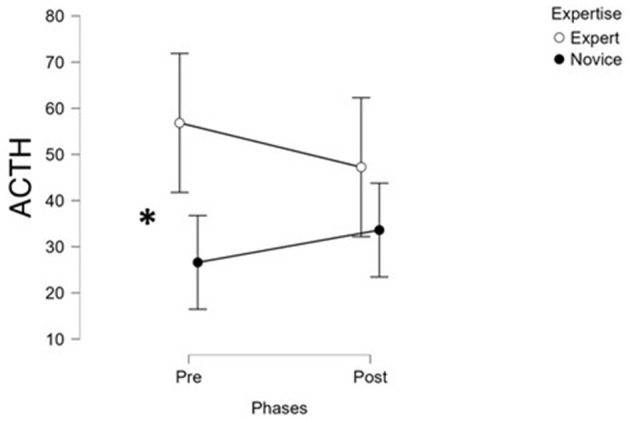
The statistical analysis performed on the ACTH biological parameter revealed a significant increase in post-surgery for Novice group, and the opposite trend for the Experts one. *Indicates statistical significant comparisons.

In contrast, ACTH levels displayed divergent trends between the two groups: ACTH decreased significantly in the Expert surgeon's post-surgery (*T*1), while it significantly increased in the Novice group (*F*(1, 30) = 8.12, *p* = 0.008, η^2^ = 0.21; Supplementary Table 1; Figure 5). This suggests a sustained or heightened HPA axis response in Novices, potentially reflecting greater physiological stress or a prolonged recovery period.

Furthermore, a positive and significant correlation was observed between the EEG-derived stress index and ACTH levels (*R* = 0.67, *p* < 10^−3^; Supplementary Figure 3), supporting the consistency between neurophysiological and endocrine measures of stress during surgery. Prolactin and metanephrines evaluation (i.e., the exploratory analytes) did not show statistically significant differences in pre- and/or post-surgery measurements.

## 4 Discussion

### 4.1 Overall results

The primary objective of this study was to provide a comprehensive characterization of stress dynamics in professional surgeons operating in a real surgical environment, using a multimodal approach that combined neurophysiological, autonomic, biological, subjective, and behavioral measures. The results offer a clear and coherent picture of how stress manifested across roles, experience levels, and surgical phases. In line with our first goal, i.e., quantifying EEG- and EDA-derived stress indices across the surgical phases, we observed that the EEG-derived stress index, based on parietal beta activity, was consistently higher in Novice surgeons compared to Experts throughout the surgery procedures, with particularly significant differences emerging during Phase 3, that is when requiring the highest level of team cooperation (Figure 2). This suggests that Novice surgeons experienced greater neurocognitive strain during the most demanding phase of the surgery. Furthermore, a detailed role-based analysis (Figure 2) revealed that this elevated stress was most pronounced in Novice first surgeons (*S*), with a similar though weaker trend for first assistants (*A*1). The EDA-derived stress index (Figure 3), reflecting autonomic arousal, also indicated higher stress levels in Novices, particularly during Phase 2. However, this autonomic marker appeared less sensitive to role-specific dynamics and phase transitions compared to the EEG-derived index (Figure 2).

With respect to the second objective, i.e., evaluating pre- and post-surgery changes in biological markers (ACTH, cortisol), our results confirmed significant reductions in cortisol levels post-surgery in both Expert and Novice groups, consistent with a generalized resolution of acute HPA axis activation (Figure 4). ACTH levels, however, revealed a distinct pattern: they significantly decreased post-surgery in Experts, while increasing in Novices. This finding points to a more sustained or possibly delayed stress response in Novice surgeons, highlighting their different physiological stress regulation compared to more experienced personnel. No differences were found in prolactin and metanephrine levels between pre- and post-surgery evaluations. This result is in line with recent studies questioning their role in response to acute stress (Matsuo et al., 2023).

The third objective, i.e., comparing stress profiles across roles and experience levels to identify groups at greater risk, was addressed by the clear evidence that Novice surgeons, particularly those in the role of first surgeon (i.e., *S*), exhibited the highest neurophysiological stress levels (Figure 2). The Novice teams also tended to report higher subjective stress and were associated with greater patient-reported discomfort, while Experts demonstrated significantly better behavioral performance as reflected in the CBTI.

Finally, the study successfully addressed the fourth objective, i.e., validating the multimodal approach through cross-modality correlations. Significant positive correlations were found between the EEG-derived stress index and both subjective stress perception and ACTH levels, as well as between EEG and EDA indices (Figure 3). This reinforces the robustness of the multimodal stress assessment framework and confirms that neurophysiological, autonomic, and endocrine markers captured coherent and temporally aligned stress dynamics.

Overall, the results of this study provided a detailed and integrative understanding of how stress develops and varies within the surgical team, depending on role, expertise, and surgical phase. They also highlight the added value of combining multi-method (physiological and biological) for a more complete assessment of stress in high-stakes professional contexts.

### 4.2 Stress indexes comparison

The multi-method approach adopted in this study allowed for an in-depth comparison of the reliability and sensitiveness of the different stress indices in discriminating levels of expertise and experimental conditions. Among all indices, the EEG-derived stress index demonstrated the highest degree of sensitivity and reliability (Figure 2). In fact, it consistently captured significant differences between Novice and Expert surgeons and reflected role-specific stress variations with temporal resolution (i.e., 1 s) that aligned well with the dynamics of the surgical task. Its ability to detect subtle fluctuations during specific phases and roles highlighted its value as a fine-grained indicator of cognitive and mental load under real conditions. The EDA-derived stress index (Figure 3), while effective in identifying overall differences in autonomic arousal between expertise groups, exhibited lower discriminative power in differentiating stress patterns. This suggested that although EDA is a reliable marker of general arousal, it may lack the granularity needed to capture the nuanced variations in stress linked to task complexity or hierarchical role during surgery. The biological markers, particularly ACTH, provided meaningful differentiation between groups, with clear post-surgery trends that contrasted Experts and Novices (Figure 5). ACTH proved more sensitive than cortisol in distinguishing sustained or heightened endocrine stress responses in Novices. However, the limited temporal resolution inherent to pre- and post-surgery sampling reduced the ability of these markers to track rapid or phase-specific stress dynamics. Subjective measures, including self-reports and SME evaluations, showed directional coherence with physiological markers but displayed limited sensitivity in detecting statistically significant differences between groups. Their reliance on personal perception or observer judgment introduced variability that can mask finer distinctions in stress response, underscoring the importance of complementing subjective assessments with objective indices. Finally, the behavioral index (CBTI) effectively discriminated between Expert and Novice teams in terms of overall performance but was not designed to measure directly internal stress levels. Its utility was to provide an external reflection of how stress may impact teamwork and efficiency rather than in sensitively detecting internal stress states.

In summary, the EEG-based index emerged as the most reliable and sensitive tool for discriminating between expertise levels and experimental conditions. While EDA and ACTH offered valuable complementary data on autonomic and endocrine responses respectively, the EEG metric provided the most granular insight into stress dynamics. The strong correlations between neurophysiological, endocrine, and subjective stress measures validate this multimodal assessment, confirming its robustness in capturing the multifaceted nature of surgical stress. This integrated approach provided empirical support for the long-held assumption that surgical experience modulates stress reactivity. Specifically, it revealed distinct, phase-specific stress patterns, highlighting the heightened neurophysiological and hormonal responses in Novice surgeons during critical stages of the procedure. The finding that Novice lead surgeons experience a particularly high cognitive load during the final, most demanding surgical phase underscores the need for targeted support and supervision during key intraoperative moments. By establishing EEG-derived metrics as the most sensitive and temporally precise indicators of intraoperative stress, this study lays the groundwork for future applications in real-time monitoring and feedback systems.

### 4.3 Limitations

While the present study provided a comprehensive and ecologically valid assessment of stress in professional surgeons during real surgical procedures, several limitations should be acknowledged. Firstly, the focus on a single type of surgical procedure, i.e., inguinal hernia repair, was chosen to standardize the task and ensure comparability across teams. However, this aspect limits the extrapolation of findings to other types of surgery, particularly those involving higher complexity, longer duration, or emergency contexts. Expanding the investigation to a broader range of procedures could provide a more reliable understanding of stress dynamics across surgical practice. Secondly, although the study employed a multimodal stress assessment, only pre- and post-surgery biological samples were collected, limiting the temporal resolution of hormonal dynamics compared to the continuous neurophysiological and autonomic monitoring. Future work could explore more frequent or continuous sampling of hormonal markers (e.g., via wearable sensors or micro-sampling techniques) to better capture the real-time interplay between endocrine and neural stress responses. A key limitation is that each surgical team performed only one procedure. While this allowed for ecological validity and adherence to OR scheduling constraints, it prevented longitudinal intra-individual comparisons. Future research should include repeated procedures for each surgeon to capture within-subject stress dynamics. Another important limitation concerns the supervision dynamics within Novice teams. In these teams, the primary operator performed the procedure under the direct supervision of an attending surgeon. This configuration may introduce a potential confounding factor, as the supervisor's presence could either buffer or increase stress depending on the interpersonal and contextual dynamics of the surgical session. Importantly, this potential bias—if present—would systematically affect all Novice surgeons in a comparable way, rather than selectively influencing only some participants. Moreover, this supervisory structure faithfully reflects real-world surgical training conditions, in which residents routinely operate under the observation and evaluation of more experienced surgeons. As such, although this factor may contribute to elevated stress in Novices, it also represents an ecologically valid component of the training environment that our study aimed to capture and quantify. Although phase- and role-specific analyses were performed, they should be considered exploratory given the limited number of teams and the multiple comparisons. Future studies with larger samples and repeated procedures will allow confirmatory modeling of phase-by-expertise interactions.

### 4.4 Future trends

The results of this study paved the way for several promising directions in future research and practical applications. One key avenue is the extension of multimodal stress monitoring to a wider range of surgical procedures, including more complex, longer-duration, or emergency surgeries. Such investigations could help to determine how stress profiles vary with surgical complexity, urgency, and unpredictability, and identify specific stressors in different operative contexts. Another important future trend involves the development of real-time stress monitoring systems integrated within the surgical environment. By leveraging wearable neurophysiological sensors, combined with machine learning algorithms trained on multimodal data, it would be possible to provide surgeons and teams with immediate feedback on their stress levels. Such systems could inform adaptive interventions, for example, pausing for microbreaks or adjusting team communication strategies to mitigate excessive stress and optimize performance during critical phases. Further, future studies should explore the longitudinal impact of stress on surgeon well-being, learning, and performance over time. Tracking stress patterns across repeated procedures could shed light on how stress evolves with experience and how it interacts with fatigue, burnout, and surgical outcomes. This would support the design of personalized stress management programs and resilience training tailored to different roles and career stages. The integration of continuous or minimally invasive hormonal monitoring technologies, such as microfluidic devices or salivary biosensors, represents another promising direction. These could complement neurophysiological and autonomic measures, enabling a more granular and dynamic assessment of the neuroendocrine stress response in real time. Finally, our findings highlighted the need for stress-adaptive surgical training platforms, including virtual reality (VR) and augmented reality (AR) simulators enriched with multimodal stress feedback (Ronca et al., 2025b; Marín-Morales et al., 2021). These tools could help trainees not only acquire technical skills but also to develop coping strategies for stress management in high-stakes environments, ultimately enhancing both performance and safety (Borghini et al., 2022a, 2024; Ronca et al., 2023, 2024b, 2025c; Sciaraffa et al., 2022b; Giorgi et al., 2023a,b; Capotorto et al., 2024). Additionally, the present findings deepen the understanding of stress dynamics in surgery and highlighted the potential for integrating objective stress monitoring into surgical education, team training, and performance evaluation frameworks.

## 5 Conclusion

The present study provides one of the most comprehensive multimodal investigations of stress in professional surgeons performing real surgical procedures, integrating neurophysiological, autonomic, biological, subjective, and behavioral measures within a single experimental framework. While previous studies have examined individual components of the stress response in surgical or simulated settings, the current work uniquely combines these complementary modalities under real operating room conditions, thereby offering an ecologically valid and temporally detailed understanding of stress dynamics in surgical teams. By examining how stress unfolds across surgical phases, roles, and levels of expertise, we demonstrated that Novice surgeons experience significantly higher stress compared to Experts, particularly during the most demanding phases of the surgery. The divergence in ACTH dynamics between Novices and Experts further highlighted differences in endocrine stress regulation, with Novices showing a sustained or heightened post-surgery stress response. Importantly, the positive correlations observed between neurophysiological, autonomic, and endocrine markers—as well as with subjective stress perception—validated the strength of a multimodal approach in capturing the complex dynamics of stress in high-stakes environments. These findings have important implications for surgical practice, training, and safety. They highlight the need for stress-aware monitoring and support systems that can identify individuals or situations at greater risk of stress overload, enabling timely interventions to optimize performance and safeguard well-being. Moreover, this work lays the foundation for future research exploring real-time stress adaptation tools and long-term stress management strategies in surgical and other high-risk professional contexts. Ultimately, by advancing our understanding of stress in the operating room, this study contributes to the broader goal of enhancing surgical outcomes, team dynamics, and healthcare quality.

## Data Availability

The raw data presented in this article might be shared upon reasonable request to the corresponding author. Requests to access the datasets should be directed to vincenzo.ronca@uniroma1.it.

## Supplementary material

Supplementary Figure 1 Repeated measure correlation analysis performed between the EEG-based stress index and the subjective stress perception.

Supplementary Figure 2 Repeated measure correlation analysis performed between the EEG-based stress index and the EDA-based stress index.

Supplementary Figure 3 Repeated measure correlation analysis performed between the EEG-based stress index and the ACTH biological parameter.

Supplementary Table 1 Values marked with ^*^ are statistically significant after False Discovery Rate (FDR) correction (Benjamini–Hochberg). Effect sizes are reported as η^2^ for ANOVA and r for *t*-tests. *CI* = 95 % confidence interval of mean difference between groups. *N* = number of participants per group (Experts = 16, Novices = 16).
