# Supplementary figures and images for: Multi-method characterization of neurophysiological and biological stress responses in surgical teams during real surgical procedures
Source: Front Neuroergon. 2026 Feb 18;7:1702748. doi: 10.3389/fnrgo.2026.1702748 (PMC12957276; doi:10.3389/fnrgo.2026.1702748)

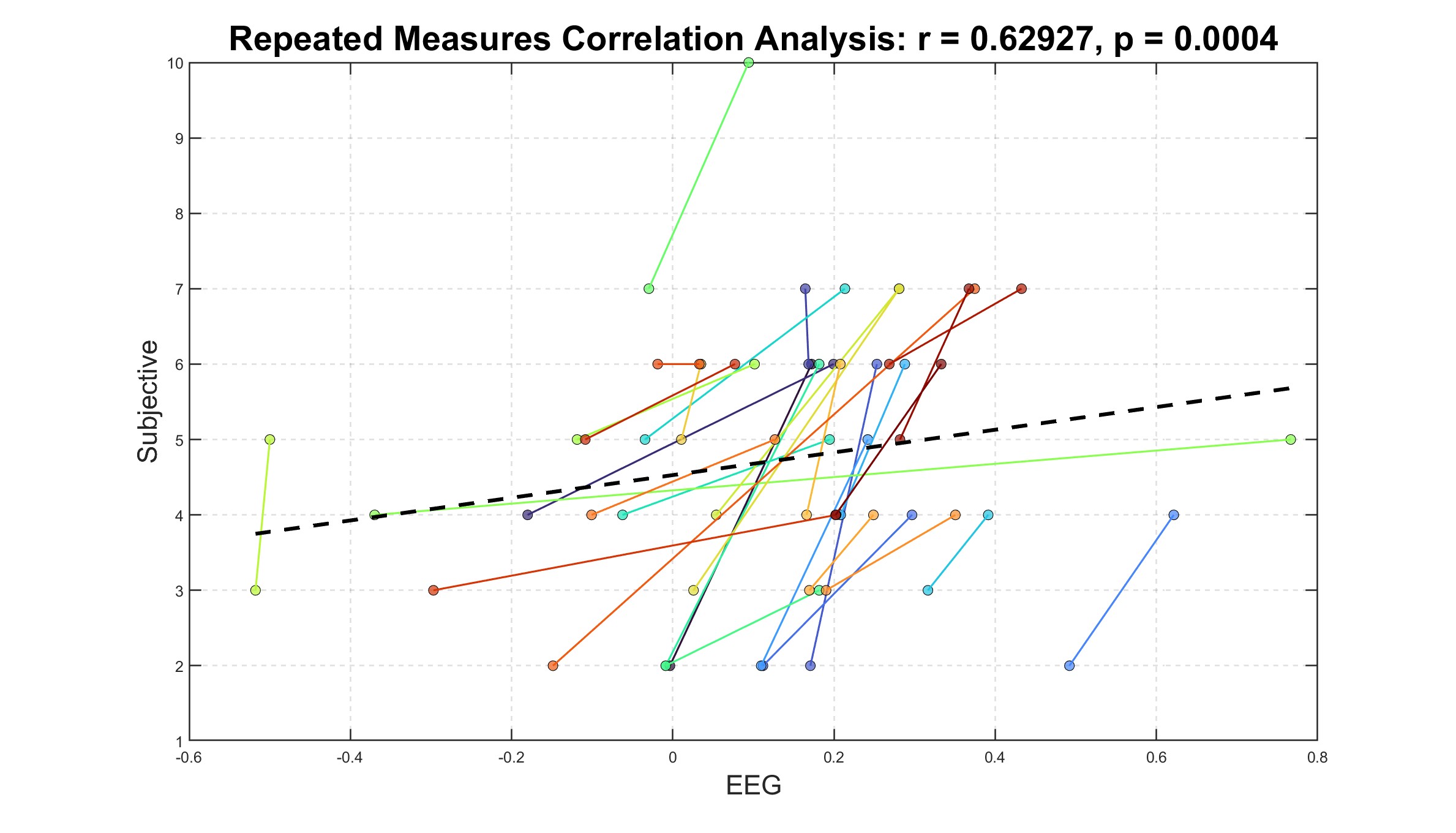

Supplement: Supplementary Figure 1 — Repeated measure correlation analysis performed between the EEG-based stress index and the subjective stress perception. [file Image_1.jpeg]

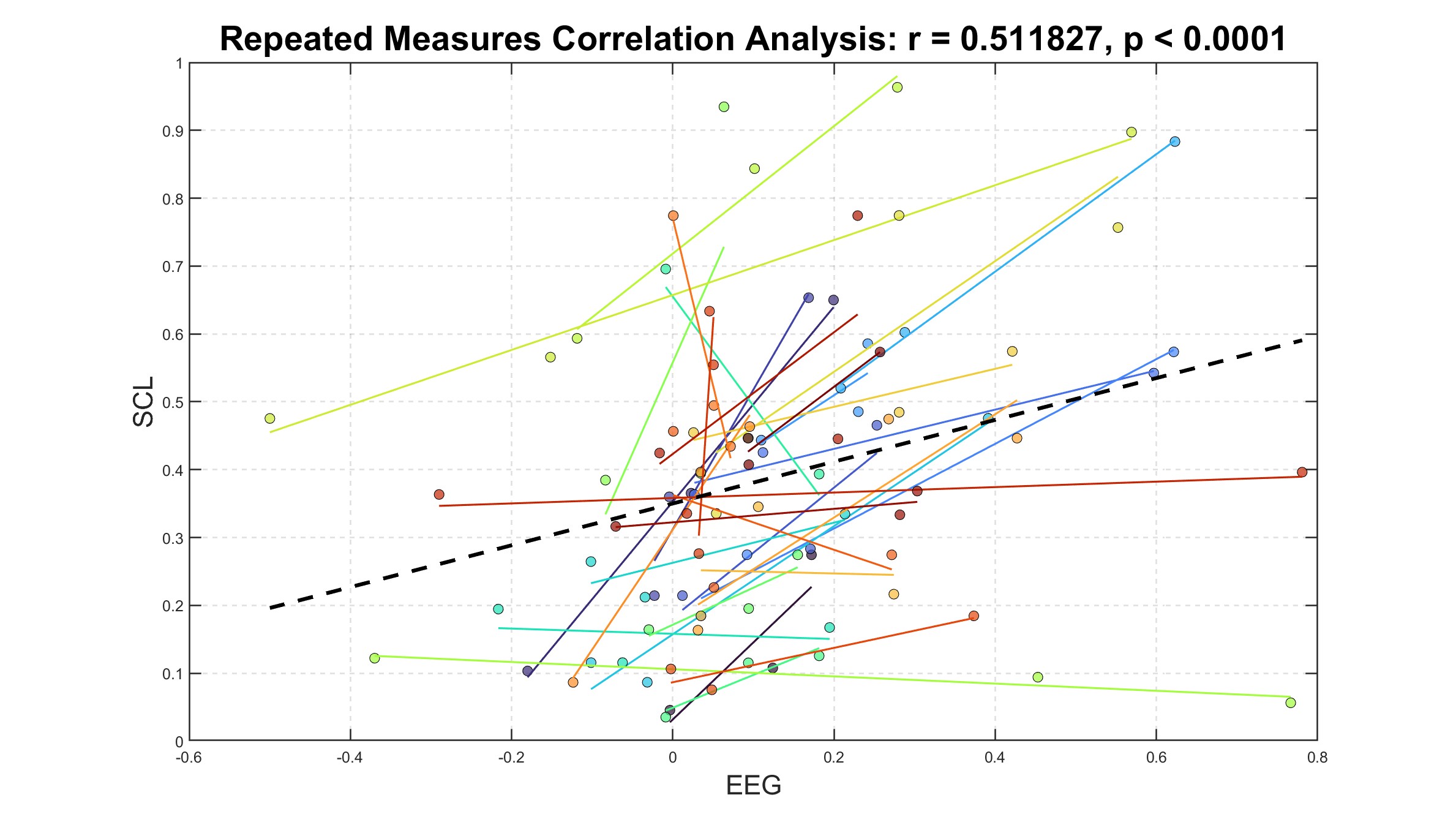

Supplement: Supplementary Figure 2 — Repeated measure correlation analysis performed between the EEG-based stress index and the EDA-based stress index. [file Image_2.jpeg]

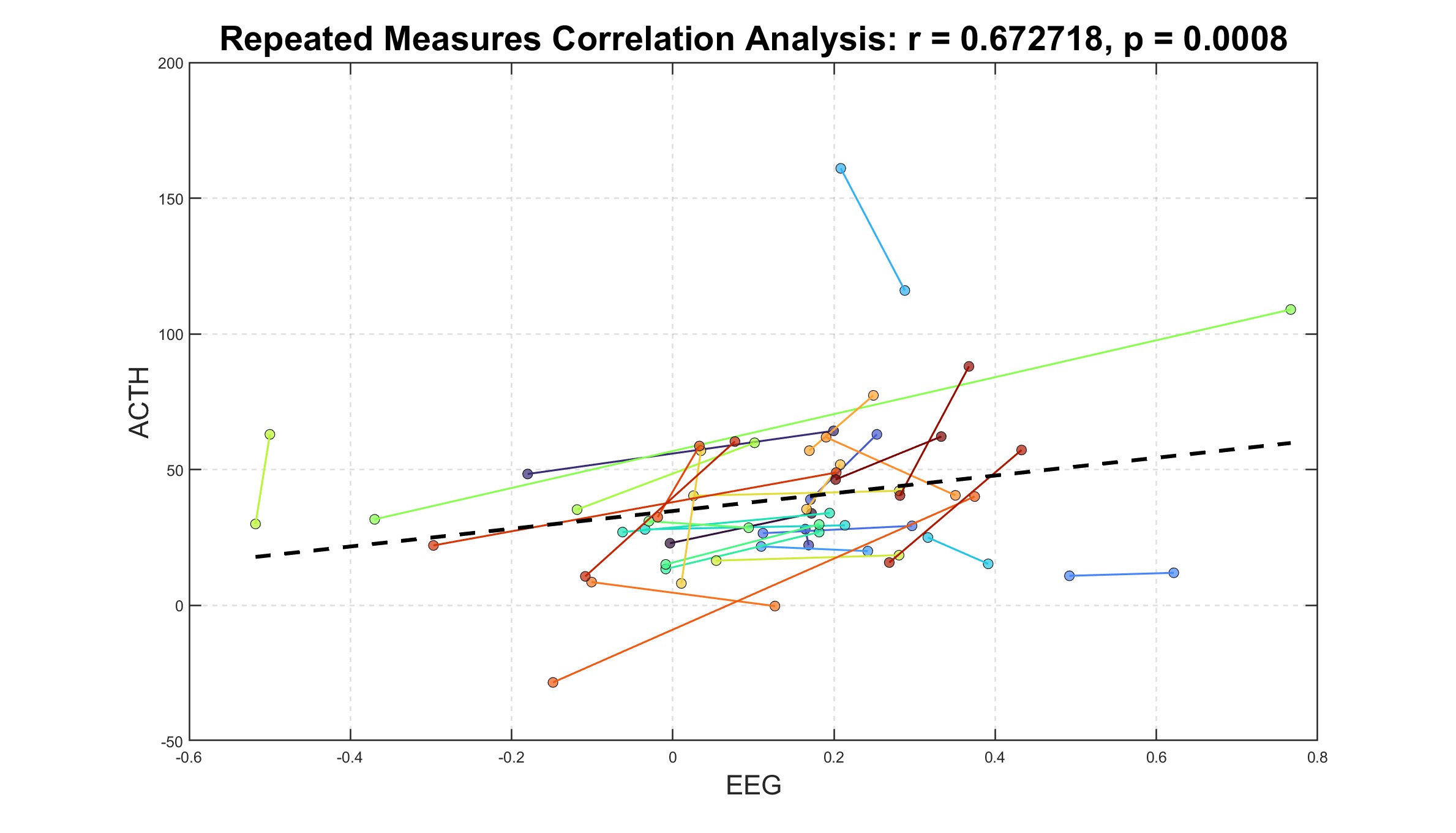

Supplement: Supplementary Figure 3 — Repeated measure correlation analysis performed between the EEG-based stress index and the ACTH biological parameter. [file Image_3.jpeg]
