# Supplementary material for: Multi-method characterization of neurophysiological and biological stress responses in surgical teams during real surgical procedures
Source: Front Neuroergon. 2026 Feb 18;7:1702748. doi: 10.3389/fnrgo.2026.1702748 (PMC12957276; doi:10.3389/fnrgo.2026.1702748)
Supplement: Supplementary Table 1 — Values marked with * are statistically significant after False Discovery Rate (FDR) correction (Benjamini–Hochberg). Effect sizes are reported as η2 for ANOVA and r for t-tests. CI = 95 % confidence interval of mean difference between groups. N = number of participants per group (Experts = 16, Novices = 16). [file Data_Sheet_1.docx]

Supplementary Table 1

Values marked with * are statistically significant after False Discovery Rate (FDR) correction (Benjamini–Hochberg).
Effect sizes are reported as η² for ANOVA and r for t-tests.
CI = 95 % confidence interval of mean difference between groups.
N = number of participants per group (Experts = 16, Novices = 16).

| **Measure / Comparison** | **Phase** | **Group difference (Experts vs Novices)** | **Test (df)** | **p-value (FDR-corr.)** | **Effect size** | **95 % CI of difference** | **N (per group)** |
| --- | --- | --- | --- | --- | --- | --- | --- |
| **EEG-based Stress Index** | Overall (all phases) | Higher in Novices | ANOVA (F = 8.21; df = 1,30) | **0.008 *** | η² = 0.21 | [0.05, 0.34] | 16 / 16 |
| EEG-based Stress Index | Phase 3 | Higher in Novices | post-hoc t (30) = 2.72 | **0.010 *** | r = 0.45 | [0.12, 0.72] | 16 / 16 |
| EEG-based Stress Index | Role: First Surgeon (S) | Higher in Novices | ANOVA (F = 7.55; df = 1,14) | **0.011 *** | η² = 0.35 | [0.08, 0.60] | 8 / 8 |
| **EDA-based Stress Index** | Overall | Higher in Novices | ANOVA (F = 5.87; df = 1,30) | **0.020 *** | η² = 0.16 | [0.03, 0.29] | 16 / 16 |
| EDA-based Stress Index | Phase 2 | Higher in Novices | t (30) = 2.32 | **0.029 *** | r = 0.39 | [0.06, 0.67] | 16 / 16 |
| **ACTH (pre → post)** | Group × Time interaction | Opposite trends (↑ Novices, ↓ Experts) | ANOVA (F = 8.12; df = 1,30) | **0.008 *** | η² = 0.21 | [0.05, 0.34] | 16 / 16 |
| **Cortisol (pre → post)** | Both groups | Decrease post-surgery | paired t (31) = 2.89 | **0.007 *** | r = 0.46 | [0.13, 0.71] | 16 / 16 |
| **Subjective Stress** | Post-surgery | Trend ↑ in Novices | t (30) = 1.81 | 0.074 | r = 0.31 | [–0.05, 0.64] | 16 / 16 |
| **CBTI** | Overall | Higher in Experts | t (6) = 2.89 | **0.021 *** | r = 0.76 | [0.13, 0.71] | 4 / 4 |
